# Postmortem imaging as a complementary tool for the investigation of cardiac death

**DOI:** 10.1080/20961790.2019.1630944

**Published:** 2019-08-19

**Authors:** Katarzyna Michaud, Pia Genet, Sara Sabatasso, Silke Grabherr

**Affiliations:** a Lausanne University Hospital and University of Lausanne, University Center of Legal Medicine Lausanne-Geneva, Lausanne, Switzerland;; b Geneva University Hospital, University Center of Legal Medicine Lausanne-Geneva, Geneva, Switzerland

**Keywords:** Forensic sciences, forensic pathology, sudden cardiac death, postmortem imaging, postmortem CT-angiography, minimally invasive autopsy, autopsy

## Abstract

In the past 2 decades, modern radiological methods, such as multiple detector computed tomography (MDCT), MDCT-angiography, and cardiac magnetic resonance imaging (MRI) were introduced into postmortem practice for investigation of sudden death (SD), including cases of sudden cardiac death (SCD). In forensic cases, the underlying cause of SD is most frequently cardiovascular with coronary atherosclerotic disease as the leading cause. There are many controversies about the role of postmortem imaging in establishing the cause of death and especially the value of minimally invasive autopsy techniques. This paper discusses the state of the art for postmortem radiological evaluation of the heart compared to classical postmortem examination, especially in cases of SCD. In SCD cases, postmortem CT is helpful to estimate the heart size and to visualize haemopericardium and calcified plaques and valves, as well as to identify and locate cardiovascular devices. Angiographic methods are useful to provide a detailed view of the coronary arteries and to analyse them, especially regarding the extent and location of stenosis and obstruction. In postsurgical cases, it allows verification and documentation of the patency of stents and bypass grafts before opening the body. Postmortem MRI is used to investigate soft tissues such as the myocardium, but images are susceptible to postmortem changes and further work is necessary to increase the understanding of these radiological aspects, especially of the ischemic myocardium. In postsurgery cases, the value of postmortem imaging of the heart is reportedly for the diagnostic and documentation purposes. The implementation of new imaging methods into routine postmortem practice is challenging, as it requires not only an investment in equipment but, more importantly, investment in the expertise of interpreting the images. Once those requirements are implemented, however, they bring great advantages in investigating cases of SCD, as they allow documentation of the body, orientation of sampling for further analyses and gathering of other information that cannot be obtained by conventional autopsy such as a complete visualization of the vascular system using postmortem angiography.

**Key points**
- There are no established guidelines for the interpretation of postmortem imaging examination of the heart
- At present, postmortem imaging methods are considered as less accurate than the autopsy for cardiac deaths
- Postmortem imaging is useful as a complementary tool for cardiac deaths
- There is still a need to validate postmortem imaging in cardiac deaths by comparing with autopsy findings

## Introduction

Cardiovascular pathologies are the most frequent causes of death in developed countries. The autopsy is widely considered as the “gold standard” in determining the cause of sudden death (SD) or sudden cardiac death (SCD). SD has been defined as “a natural, unexpected fatal event occurring within 1 h from the onset of symptoms in an apparently healthy subject or whose disease was not so severe as to predict an abrupt outcome” [1] and SCD is defined as an SD resulting from cardiac causes. The autopsy allows detection of hereditary forms of cardiovascular pathologies, allowing notification and intervention in relatives at risk [2]. Radiological examination has been used in forensic and clinical pathology in specific situations of SD for many years, as an additional diagnostic tool, for documentation and for research, mainly in an academic setting. More recently, tomographic and other forms of digital imaging have been introduced, first as whole-body imaging by postmortem computed tomography (PMCT) and then by postmortem magnetic resonance (PMMR) imaging, with or without additional angiographic methodology [3–6]. Today, PMCT remains the most widely accessible and the most frequently used of these methods, though limitations of PMCT in natural deaths and especially in cardiovascular pathologies have been reported [7]. There are still many controversies about the role of postmortem imaging, and whole body imaging methodologies are considered less accurate than the autopsy for sudden natural deaths [7–11].

Many reference sources explain how to dissect the heart at autopsy and how to perform sampling for histological examination. The possibilities of visualizing different cardiac structures, including the fibrous endoskeleton of the heart, have been investigated [12,13]. Clinical guidelines set in the fields of medical imaging and cardiology allow standardized interpretations of cardiovascular imaging data. However, there are no established guidelines for the interpretation of postmortem imaging examinations of the heart. The goal of this paper is to present the state of the art of postmortem imaging evaluation of the heart compared to the classical postmortem examination, especially in cases of SCD.

## Postmortem imaging methods used for cardiac examination

In recent years, imaging methods have become prominent in postmortem investigations, especially in forensic medicine [5,14,15]. Centres all over the world have started to apply imaging techniques in order to enhance the quality of the postmortem examination and to document the investigated body digitally. The advantages and limitations of the imaging examination vary with the technique applied. CT methodologies offer advantages in investigating traumatic death [16], as they are most appropriate to visualize the skeletal system and gas/fluid spaces as well as (mostly “solid”) foreign bodies. Therefore, the detection of bone fractures is rendered possible. The three-dimensional (3D) demonstration of complex fracture patterns is of highest interest in forensic investigation of traumatic death. In modern forensic imaging, basic and complementary (minimally invasive) methods can be employed as needed [17,18].

### Basic methods

The most often applied basic methods in postmortem investigations are conventional radiography (CR), PMCT and PMMR.

#### CR

The idea of using X-rays for medicolegal purposes is nearly as old as radiography itself. In fact, only 1 year after the discovery of X-rays by Wilhelm Conrad Röntgen in 1895 [19], the mummified hand of an Egyptian princess was subjected to radiography. CR is still used regularly in postmortem investigations, especially in forensic medicine, as it allows the rapid detection of foreign bodies such as projectiles which are of utmost importance in forensic investigations. Foreign bodies such as prosthetic materials are extremely useful to identify persons.

One of the main disadvantages of CR is the fact that soft tissue is visualized in a very limited way. No detailed images of organ parenchyma are obtainable [17], which renders its application to the investigation of natural death extremely limited. The use of plain radiography for investigating the heart is mainly useful in the detection of radiopaque foreign bodies such as radiopaque stents, or to grossly assess the shape of the heart. The so-called cardio-thoracic ratio (CTR) is of use in order to identify cardiomegaly on PMCT [20].

#### PMCT

PMCT is the most frequently used technique in modern forensic imaging. To our knowledge, its first application for forensic purposes was reported in 1983 [21]. Since that time, its advantages and limitations have been investigated in multiple studies [21–23] and its role as an additional tool to conventional autopsy is uncontested today. Many centres of legal medicine have been equipped with a CT scanner and the performance of a PMCT of the investigated body has become a routine investigation in many countries. Even centres, which do not have their own equipment, perform PMCT by using the scanners in the radiology department. PMCT’s most important advantages are again seen in the investigation of traumatic death. In fact, as a method based on X-ray technology, it is mostly suited to visualize bone structures and air. Outside of its ability to visualize coronary and other calcifications, it is limited for the investigation of SCD. However, it can give information about ischemic heart disease (IHD). Although no information can be gained concerning the degree of a stenosis and the presence of ischemic lesions in the myocardium, such a suspicion can be used as indication for further examinations such as postmortem computed tomographic angiography (PMCTA).

#### PMMR

PMMR is a technique that does not use any radiation [24]. Its physical principle is based on use of the magnetic properties of certain atomic nuclei. After stimulation by magnetic fields and radiofrequency, specific signals are emitted which can be transformed into images [17,18]. MRI is especially useful to investigate soft tissue. This renders this method potentially useful for investigating the heart and, therefore, cases of cardiac death. Despite these evident advantages, PMMR is not yet established in postmortem investigations [22]. This can be explained by the fact that PMMR has important limitations, including long examination times and high costs. PMMR technology is very complex, requiring specially trained personal for performing the exams and for interpreting the obtained images. Also, PMMR is highly sensitive to temperature and the influence of decreased body temperature in deceased is not completely understood [3,23,25].

Once these problems can be overcome, PMMR is probably the best choice to investigate the heart and can give extremely useful information about the myocardium. T1- and T2-weighted sequences supply anatomical information and can highlight fluid accumulations such as oedema, closed collections such as cysts and abscesses, haemorrhage, organ lacerations, contusions and effusions. It is especially indicated for investigating myocardium [26]. In fact, it can depict acute myocardial infarction by showing focal necrosis surrounded by oedema, which is visible on T2-weighted images. A decrease in signal intensity within the myocardium is consistent with early acute myocardial infarction [26].

### The complements

Minimally invasive interventions, which allow the performance of postmortem angiography (PMA) by injecting a contrast agent, can be accomplished with PMCT and PMMR. This allows image-guided sampling of tissue or liquid aspiration. Mostly, PMCT is used to perform such interventions.

#### PMA

By injecting a contrast agent into a vessel, the vessel’s lumen can be visualized, allowing the identification and quantification of stenosis. PMCTA is used to acquire the imaging data, which can be formatted into 3D-reconstructions of the vascular system. Different methods of PMCTA exist to allow whole-body and targeted angiography [5,27]. There is a range of contrast agents and administration techniques for different applications, including simple manual injection or the use of injection devices [5,28].

The most widespread method of whole-body angiography is multiphase postmortem computed tomography angiography (MPMCTA) developed by Grabherr et al. [29]. By applying this standardized technique, the vasculature of the head, thorax, and abdomen can be visualized by using the femoral vessels for access. The advantages of this technique over conventional autopsy have been investigated by multiple studies [6,30]. As it allows to investigate the coronary arteries and even gives some information about the myocardium, it is especially useful for investigating SCD [3,5,9,31]. Targeted coronary artery methods [32,33] allow detailed visualization of the coronary arteries by selected filling of the ascending aorta and the coronary arteries through the insertion of a catheter via a carotid artery [5]. A recent study has hypothesized that this technique could avoid invasive autopsy in cases of cardiac death [34].

#### Postmortem tissue sampling

In order to obtain samples from the body without using a standard conventional autopsy, image-guided tissue sampling or needle aspiration can be performed. This technique is often called “minimally invasive autopsy” (MIA) [35]. PMCT is usually the technique used to obtain samples from specific organs and tissues for histological examination [36]. Beside liquid samples for toxicology, microbiology etc., and histological samples, gas can be sampled from the body in order to allow analysis of its composition [37]. This can be useful to differentiate vital air embolism from postmortem gas collections [38] or to investigate scuba diving accidents [39]. Extensive protocols for obtaining different samples are available in cases when PMA should be performed [5]. Minimally invasive sampling before injecting a contrast agent can avoid alteration of analyses due to the perfusion liquid.

## Radiologic examination of the heart

### Heart weight and size

The heart weight and size at autopsy and their comparison to predicted values are one of the first and most important steps to evaluate the heart. An increased heart weight and size is observed in some cardiomyopathies, such as hypertrophic cardiomyopathy, in remodelled hearts in advanced IHD, and in some valvular diseases. The heart is weighted at autopsy after being emptied of blood clots, the wall thickness is assessed, and the transverse and longitudinal dimensions are measured [2].

In postmortem practice, the CTR value was first evaluated on PMCT by Winklhofer et al. [40], who suggested a threshold of 0.57 as a cut-off of CTR to detect cardiomegaly with a specificity of 95%. Jotterand et al. [20] evaluated the CTR on PMCT for normal hearts considering the normal reference values for the heart weight and observed an overestimation of the CTR measurements made on scout compared to axial view. It was also shown that among the different variables tested, body mass index (BMI) and heart dilatation influence the postmortem CTR [20]. The role of dilatation was not evaluated by Winklhofer et al. [40] while cardiac dilatation and terminal central congestion was reported by Michiue et al. [41] in cases of drowning, alcohol/sedative-hypnotic intoxication, and some SCDs. Jotterand [42] presented at the International Society of Forensic Radiology and Imaging meeting in 2017 a “new formula for CTR for the diagnostic of cardiomegaly on postmortem CT”. This study proposed a formula of adjusted CTR to predict “cardiomegaly” on PMCT. It was shown that CTR alone cannot be used to discriminate between normal and increased heart weight at PMCT and that factors associated with an increased heart weight are CTR, BMI, age and gender (under review). Jakobsen et al. [43] in 2016 showed that a non-invasive *in situ* PMCT method for organ measurement is not useful as no correlation were found between *in situ* and *ex vivo* volumes of the heart. However, a highly significant correlation was found between the *ex vivo* volumes and weights of the heart. PMCT-determined CTR was considered useless for ascertaining cardiomegaly, as it neither correlated with the *ex vivo* heart volume nor with the heart weight-ratio based on the comparison of the heart weight at autopsy and estimated upper limit of a normal heart.

Cardiac MR is considered the gold standard to determine the left ventricular mass in living patients [44]. Some studies were performed also with PMMR; in 2014, Ruder et al. [23] suggested that single circumferential area measurements of the left ventricle in the four chamber view on cardiac PMMR reflects the heart weight and proposed a simplified formula for heart weight derived from linear regression analysis. Prodhomme et al. [45] studied 16 cases of sudden unexpected death in infancy where they investigated semiautomated 3D volume reconstruction method for organ volume measurement by postmortem MR and concluded that this method may provide significant information concerning the macroscopic features of the main organs. However, this technique was considered as least useful for the adrenals and the heart.

### Coronary arteries

Evaluation of the coronary arteries is one of the essential steps in postmortem investigation, as many SDs are related to coronary artery lesions. SCD related to atherosclerotic coronary artery disease and to IHD is the most frequent cause of death in the general population in the Western world [46,47]. According to the updated guidelines for autopsy investigation of SCD, the death can be considered as certainly related to atherosclerotic coronary artery disease in the presence of an acute coronary occlusion (atherothrombosis, arteritis, dissection or embolism) or as highly probable in the presence of atherosclerotic plaque with coronary luminal stenosis >75% [2]. Anomalous origin of the left main coronary artery from the pulmonary trunk is also considered as a certain cause of death while an interarterial course of the left coronary artery originating from the right sinus is a highly probable cause of death.

**Figure 1.**
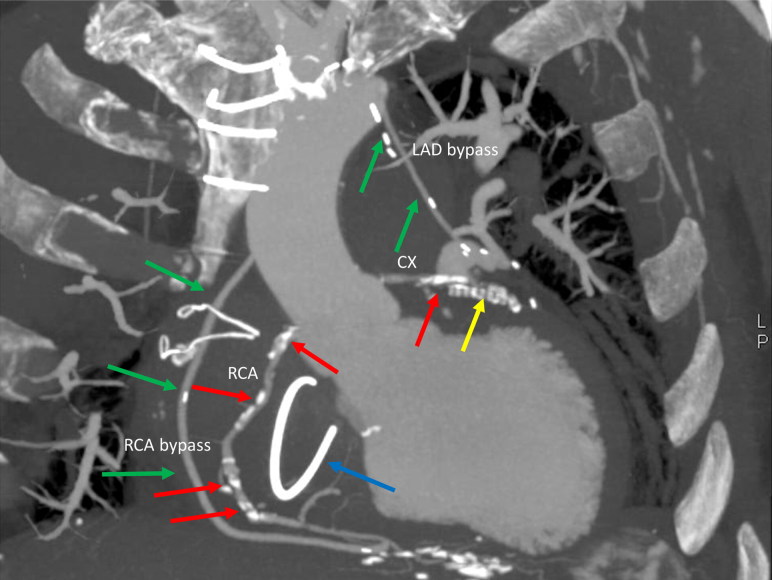
Coronal view of MPMCTA of the heart, in the arterial phase, in MIP, with visualization of calcifications of the native coronary arteries (RCA, CX, red arrows) and a post-surgery status with a stent in the CX (yellow arrow), two coronary bypasses, one bypass between the aorta and the RCA and one between the left internal mammary artery and the LAD (green arrows). A prosthesis of the tricuspid valve (blue arrow) is also noted. MPMCTA: multiphase postmortem computed tomography angiography; MIP: maximum intensity projection; RCA: right coronary artery; CX: circumflex coronary artery; LAD: left anterior descending coronary artery.

The radiological examination of coronary arteries often gives the first appreciation of the coronary status and allows documentation before performing an autopsy (Figure 1). PMCT permits the detection of coronary artery calcifications while PMCTA additionally allows for the evaluation of coronary artery lumen [4,31,48,49] and can also detect associated myocardial lesions such as myocardial rupture, and in some cases, signs of myocardial infarction. The most important task is detection of coronary thrombosis. A specific key for postmortem radiological reading of coronary arteries has been proposed thanks to a detailed comparison between radiological images, autopsy and histology [5].

#### Coronary calcifications

Coronary artery calcium score (CACS) is an independent predictor of coronary artery disease events in clinical practice and has been found to be a marker of vascular injury that correlates closely with overall atherosclerotic burden. Traditionally, coronary calcification is quantified radiologically by Agatston’s score. The radiological grading of coronary artery disease, based on this score, is classified as no evidence of coronary artery disease (0 calcium score), minimal (1–10), mild (11–100), moderate (101–400) and severe (>400). In living patients, a zero CACS is considered the most powerful negative risk factor for development of a coronary event. It is known that about 4% of living patients present with non-calcified plaques and a zero CACS [50–52]. Therefore, forensic pathologist and radiologists should evaluate very carefully all coronaries of SD victims, even if calcifications of coronary arteries are not extensive in PMCT.

Postmortem imaging of treated coronary arteries appears especially useful and is recommended. According to the literatures [4,30,53], PMCT allows for the detection of coronary stents, which could be difficult to visualize at autopsy in the presence of severe calcifications. MPMCTA can demonstrate the patency of coronary bypass grafts and help guide the dissection technique during autopsy [2,5,49,54].

### Valves and wall thickness

During classical autopsy, valves are examined visually and measured after the opening of the heart. The ventricular thickness is measured at the mid-cavity level, excluding trabeculae. Only a few postmortem radiological examination studies discuss postmortem radiological examination of the valves. Ampanozi et al. [55] showed that several cardiac PMMR measurements differed from corresponding autopsy measurements. In a study of 50 cases comparing cardiovascular measurements for aorta, heart cavities, cardiac wall thicknesses, maximum cardiac diameter and CTR between the native CT scan (without contrast), the CT angiography and autopsy, it was shown that only aorta measurements in CT angiography correlate with measurements obtained at autopsy [56]. PMCT is the preferred technique for showing the extent of calcifications in the fibrous skeleton but there are no reports on valvular pathologies in larger postmortem series [10,13].

### Myocardium

**Figure 2.**
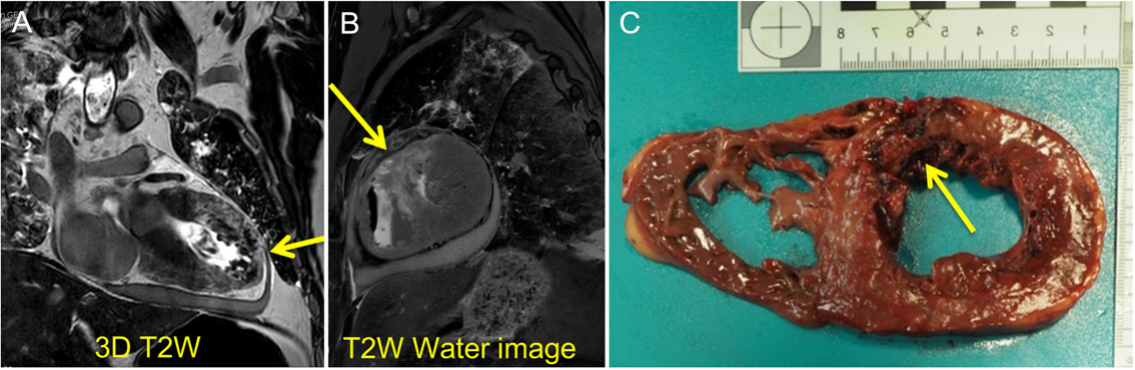
(A) coronal and (B) sagittal view of a PMMR of the heart with visualization of a hyper-intense area of the myocardium of the anterior wall of the left ventricle and the inter-ventricular septum (yellow arrows), compatible with an infarction; (C) confirmation of the myocardial infarction macroscopically visible at autopsy (yellow arrow).

PMMR is considered the method of choice for non-invasive investigation of the myocardium. Jackowski et al. [57–60] demonstrated that PMMR enables the detection of myocardial infarction *in situ* and the estimation of infarct age based on signal behaviour and quantitative T1, T2 and proton density values, better if temperature-corrected (Figure 2). Oedema was suggested as a sign of early myocardial infarction in *ex vivo* porcine hearts [61]. In 2018, Wagensveld et al. [62] found that the combination of MRI with heart biopsies had high sensitivity and specificity for the detection of acute and chronic myocardial infarction at postmortem examination. In spite of the good performance of PMMR to detect myocardial infarction/ischemia, this option remains relatively inaccessible, and forensic and clinical pathology centres with direct access to MRI are rare. On the other hand, an increasing number of these centres have access to PMCT and often to PMCTA.

Unenhanced PMCT is known to have a low diagnostic value for the detection of myocardial infarction, but PMCTA, namely MPMCTA, has recently been shown to be able to detect infarcted areas as regions of increased signal. In this sense, MPMCTA could help to identify affected regions for guiding and improving the sampling for microscopic examination [4,5,63,64]. However, few studies have been performed on the diagnostic value of MPMCTA for myocardial infarction/ischemia; therefore, further and larger investigations are needed before introducing it for this purpose as a routine procedure.

Medical images are often affected by postmortem changes and further work is necessary to increase the understanding of postmortem myocardial imaging [23,65,66].

Up to now, the diagnostic value of postmortem imaging to detect pathologies such as myocarditis and cardiomyopathies (such as hypertrophic cardiomyopathy or arrhythmogenic cardiomyopathy) has not been demonstrated [10,11].

## Artefacts in postmortem imaging

When analysing postmortem images, it is important to know and to keep in mind that there exist both imaging artefacts and postmortem artefacts, which have to be taken into account during the evaluation of postmortem data. Not considering them can lead to an incorrect interpretation of the images.

In PMCT, there exist imaging artefacts, which are based on physical characteristics, such as the partial volume effect, which occurs when image section thickness is more than object thickness, rendering it indistinct; photon starvation, streaks that become visible when thin sections through thick bone are acquired; or beam hardening, which causes streaks by lower X-ray energies being filtered out by bone. Scanned dense objects, such as metal, for example artificial valves or other implanted cardiac devices, may also cause streak artefacts, which overlay the actual image and which impede the accurate evaluation of the image. Moreover, there exist also machine-related artefacts, which can influence the interpretation of the images, such as incomplete projections (part of the object existing peripheral to the field of view) or ring (due to a miscalibrated or detective detector element, which results in rings centred on the centre of rotation) and line artefacts (due to statistical error of low photon counts) [5,67].

In cardiovascular PMCT, the most important artefacts are those that are related to postmortem changes. Flattening, or complete collapse of the vessels can be seen, as well as the presence of gas in the vascular lumen [5,68].

There exist also characteristic PMCTA findings, which are important to know to avoid misinterpretation. The most important finding is the formation of postmortem blood clots within liquid blood, which creates filling defects in the vessels [66,69,70]. Two other PMCTA findings, which are important to keep in mind are contrast layering and inhomogeneous opacification of the vessels [5]. In cardiovascular imaging, these can mimic a stenosis in the coronary artery or an embolism in the pulmonary arteries. If using MPMCTA, Grabherr et al. [5] reported that it is crucial to analyse the three phases of the multiphase-PMCTA, and not only one, to distinguish a postmortem effect from a real stenosis or embolism. However, in some cases, it is impossible to prove, based solely on postmortem imaging, if it is contrast layering/postmortem clot or a real finding, and in those cases the only possibility to differentiate between the two of them is the confirmation by autopsy and histological examination.

PMMR is an imaging method that is being employed more and more in postmortem imaging and it is therefore important to be aware of the artefacts peculiar to PMMR. The most frequent MRI-artefacts are ghost artefact due to motion of the body, chemical shift artefact due to frequency difference between adjacent fat and water, and magnetic susceptibility artefact due to other differences between tissues. Other artefacts include phase sampling artefact and truncation artefact. Moreover, there exists magnetic artefact due to an overlapping of slices which can provoke a deformation of the image and a loss of information during the acquisition.

In PMMR angiography, which is a topic of research, the same findings that are visible in PMCTA and are related to the contrast medium layering in blood vessels or revealing postmortem clot, can be observed.

## Congenital malformations

There exist several cardiovascular malformations that can be visualized in postmortem imaging. Most of the severe malformations of the cardiovascular system are already known in younger patients and postmortem imaging in this situation mostly involves neonates. However, there are also occult cardiovascular malformations that are therefore never detected during life or which present at a later age.

Most cardiovascular malformations concern the big vessels and the heart. They can be visible in form of a malposition of the coronary arteries, a transposition of the great vessels, or a stenosis of the major vessels (e.g. congenital pulmonary stenosis or aortic coarctation) [71,72]. Those malformations are recognisable in PMCT and can be well analysed with PMCTA and/or PMMR. Cardiovascular malformations also involve the heart valves (e.g. bicuspid aortic valve). PMCT is not the modality of choice for bicuspid aortic valve. With PMCTA it is also difficult or rather impossible to evaluate the heart valves in a correct manner. The only postmortem imaging method, which allows an appropriate analysis of the structure of the heart valves, is PMMR [55]. PMMR is also the method of choice for evaluating the myocardium and its thickness, like for example in cardiac hypertrophy although PMCT and PMCTA can already give a hint on the heart size and the wall thickness of the myocardium.

Patent foramen ovale and persistent truncus arteriosus can be visualized on PMCTA, as the contrast medium will pass from one atrium to the other or from one great vessel to the other, respectively [5].

PMCT, PMCTA and PMMR can be helpful tools in searching for cardiovascular malformation; especially PMMR can offer structural information which are comparable to that of conventional autopsy, especially in foetuses. As an adjuvant to conventional autopsy, cardiac postmortem MRI may increase the outcome from conventional autopsy and may play a major role in developing less invasive autopsy methods [5]. It has been shown that micro-CT of small *ex vivo* foetal specimens can provide highly accurate 3D rendering of complex congenital foetal heart disease [73]. It also has been shown that high-field PMMR, PMCT and micro-CT may allow less-invasive postmortem assessments [74].

## Postmortem imaging after cardiac surgery

The investigation of death after cardiac surgery is important for many different reasons: it helps to define the exact cause of death and may allow understanding what has happened with the patient. It also can give answers to medicolegal questions such as is there causality between the surgery and death or did the death occur for other reasons? Was a medical error made? Is the medical staff responsible? Could the death have been avoided by choosing another approach? And finally: what can be done in future to avoid the death of other patients?

As different studies show [75], death after medical interventions is often related to postoperative haemorrhage, especially if the cardiovascular system was the main focus of the surgery. Postmortem imaging can be used to assess the body immediately after death and before performing any invasive manipulation due to autopsy. Mostly, PMCT is applied in order to depict the exact location of foreign bodies, such as surgical material, clips, prostheses etc. [76]. PMA is especially indicated in such cases, as it allows visualizing an eventual source of bleeding in a way that is clearly superior to conventional autopsy [77].

PMCT and PMA are indicated in cases of death after cardiovascular surgery. The advantages of multi-phase PMCTA have been investigated and described in the literature [78]. The images obtained by this technique help to identify the cause of death, and to visualize eventual complications that may have occurred during the intervention [5].

PMA allows visualization of the bypass to control its permeability. The advantages of PMCT include exact localization of surgical clips that typically accompany the manipulated vessel [79].

After cardiac valve surgery or cardiac transplantation, sutures, clips and the position of the valves have to be examined. Complications can occur due to many causes, for example the insertions of catheters, manipulation of the implant. PMA can identify the source of bleeding that can be identified by visualizing extravasation from a ruptured coronary artery or from a perforation in the myocardium [80].

Ventricular Assist Devices (left, right or biventricular) can be visualized by native CT (without contrast), allowing the inspection of the devices and their exact localization.

In order to verify the correct position and function of a vascular prosthesis such as an aortic prosthesis, the performance of PMA is essential. In fact, using contrast, a perforation or a dissection of the vascular wall can be ruled in or out [81].

## Summary and conclusion

The use of MIA techniques has increased in recent years. Although imaging techniques are well established in clinical investigations, they cannot simply be transferred to postmortem examination. In fact, postmortem changes are evident and lead to the need for developing new competences in order to interpret the obtained images. Neither a clinical radiologist nor an expert in postmortem examination may have those competences, which have to be developed in a common approach between those two different specialties. Some examinations such as PMA require specific material and techniques in order to obtain images comparable to clinical angiographic images (Table 1) [3–5,15,17,18,23,31,54,55,78,82–87].

**Table 1.** Summary of advantages and disadvantages of various radiological methods used in postmortem heart examination.

Method	Advantages	Disadvantages	References
Traditional plain X-rays	Fast examination	Radiation	[54]
	Easy to handle	No 3D reconstructions	
	Simple data storage		
	Relatively low maintenance costs	Very limited visualization of soft tissue	
	Visualization of skeletal system	Superimposed image	
	Detection of foreign bodies	Quality strongly depending on acquisition	
	Inexpensive	Diagnostics:	
	Diagnostics: CTR Haemopericardium Major calcification of plaques and valves Identification and localisation of cardiovascular devices	Impossible to visualize the lumen of the vessels, the valves and the myocardium Low accuracy for cardiovascular pathologies Impossible to guide the biopsies	
	Fast examination	Radiation	
	Easy to handle	Data storage	
	Ideal for 3D reconstructions	Limited visualization of soft tissue, organs, vascular system	
PMCT	Relatively low maintenance costs	Training for correct interpretation needed	[15,17,30,78, 82]
	Excellent visualization of skeletal system, gasGuided biopsies possibleDiagnostics: Possibility to estimate the heart size by CTR Possibility to detect some pathologies explaining the death (e.g. haemopericardium) Calcifications of plaques and valves Identification and localisation of cardiovascular devices	Diagnostics: Limited application and low accuracy for cardiovascular pathologies Impossible to evaluate the lumen of coronary arteries, the valves and myocardium	
PMCTA	Excellent visualization of the soft tissues, the organs and the vessels	Radiation	[3–5,54]
	Relatively fast examination	Data storage	
	Ideal for 3D reconstructionsGuided biopsies possibleDiagnostics: Detailed evaluation and documentation of the distribution pattern of epicardial coronary arteries, information on extent and location of arterial stenosis/obstruction, including patency of stents and bypass grafts Detection of the origin of a haemorrhage (rupture of infarcted myocardium versus aortic dissection) Indication of suspected area for autopsies for histological examination on radiologically pathological areas	Training for correct use and interpretation is neededHistological and radiological artefactsDiagnostics: Limited interpretation of the myocardium and the valves Interpretation limited by artefacts	
PMMR	Excellent visualization of the soft tissue, organs, vascular wall	Time consuming	[18,23,83, 84]
	No radiation	More difficult to handle	
	Guided biopsies possible	High maintenance costs	
	Diagnostic:	Need specific architectural construction	
	Good visualization of the soft tissues, especially the myocardium	3D reconstructions need special sequences	
		Data storage	
		Training for correct use and interpretation is needed	
		Diagnostics:	
		Interpretation needs to consider the postmortem modifications	

		Interpretation limited by artefacts	
		Interpretation of the lumen of the coronary arteries limited	
PMMRA	Excellent visualization of the soft tissue, organs, vascular wall	Time consuming	[54,85–87]
	No radiation	More difficult to handle	
	Guided biopsies possible	High maintenance costs	
	Diagnostic:	Need specific architectural construction	
	Good visualization of the soft tissues, especially the myocardium	3D reconstructions need special sequences	
		Data storage	
		Training for correct use and interpretation is needed	
		Diagnostics:	
		Interpretation needs to consider the postmortem modification	
		Interpretation limited by artefacts	
		Interpretation of the lumen of the coronary arteries limited	

CTR: cardio-thoracic ratio; 3D: three-dimensional; PMCT: postmortem computed tomography; PMCTA: postmortem computed tomographic angiography; PMMR: postmortem magnetic resonance; PMMRA: postmortem magnetic resonance angiography.

In general, classic techniques of postmortem investigations and the new radiological techniques are complementary. According to the recent review paper of Blokker et al. [9], postmortem imaging methods are still less accurate than the autopsy [88] for natural deaths. This statement has to be interpreted carefully as the accuracy of postmortem imaging clearly depends on the technique used and the limitations of each technique should be considered. The advantages and disadvantages of various whole-body imaging methods are related to the logistics, cost, and image quality. By further developing techniques such as PMA and by increasing the quality and experience of the interpretation of the obtained images, the actual role of such imaging techniques should be re-evaluated constantly.

In conclusion, the implementation of new imaging methods into routine postmortem practice is challenging, as it needs not only an investment in equipment but also in the expertise of interpreting the images. Once those requirements are fulfilled however, they bring great advantages to the investigation of cases of SCD, as they allow documentation of the body, to select samples for histological examination from suspected areas of the heart and vessels, to identify indications for further analyses and to obtain information that cannot be obtained by conventional autopsy such as a complete visualization of the vascular system using PMA.
